# Creating a sustainable brain health navigator model to improve the diagnostic journey for Alzheimer's disease: the experience of one health care system in California

**DOI:** 10.1002/alz70860_101327

**Published:** 2025-12-23

**Authors:** Michael Young Lee, Soo Borson, Elizabeth B Joe, Jennifer Marks, Bonnie Olsen

**Affiliations:** ^1^ Keck Medical Center of USC, part of Keck Medicine of USC, Los Angelos, CA, USA; ^2^ Keck USC School of Medicine, Los Angeles, CA, USA; ^3^ Keck School of Medicine of USC, Los Angeles, CA, USA

## Abstract

**Background:**

Keck Medicine of USC (KMUSC) serves a culturally diverse population of 16 million in urban and rural Southern and Central California. In FY24, KMUSC staffed 800,000 outpatient visits encompassing a wide range of demographics and medical needs. A population‐based MCI/dementia detection and diagnosis plan is a critical step toward a fully realized Brain Health Program at KMUSC. Our model, augmented by a BHN, will identify individuals with potentially undiagnosed cognitive impairment through risk stratification and deployment of an EHR‐integrated digital cognitive assessment, and streamline, standardize and facilitate the diagnostic process. Bringing a BHN into the diagnostic process will accelerate achievement of this goal and promote future spread of our fully developed processes to satellite clinics and other disciplines.

**Method:**

An interdisciplinary clinical and IT implementation team is collaborating on the design and operational details for integrating the BHN role into primary and specialty workflows, to ensure that patients receive timely assessment, referrals, and post‐diagnostic care. Our Brain Health Navigator (BHN) pilot will launch in the USC Healthcare Center 2 with co‐located primary (Family Medicine and Internal Medicine) and specialty memory (Neurology) care services.

**Result:**

We expect to create substantial value for patients/families, their primary care clinicians, and memory specialty providers. We will choose metrics to assess the effectiveness of our BHN‐facilitated pathway in (1) increasing diagnosis of cognitive impairment, and (2) achieving timely, targeted referrals. In addition, we will explore ways to collaborate on post‐diagnosis care pathways, engaging both primary and specialty care clinicians. Because KMUSC is a “Guiding an Improved Dementia Experience” (GUIDE) program site (starting July 2025), patient/caregiver referral to GUIDE will become a useful longitudinal measure of how well the diagnostic pathway is working. The BHN, working to facilitate identification and diagnosis of MCI/dementia, will become a natural partner for the GUIDE post‐diagnosis navigator, together providing ‘wrap‐around’ support for optimal benefit to patients and families.

**Conclusion:**

The BHN Model and other resources of DAC‐SP support the implementation of boundary‐ spanning roles across primary and specialty care, designed for a timely, accurate, and effective journey through diagnosis and care across Keck Medical at USC and similar health systems.

